# Genome-Wide Identification of 109 NAC Genes and Dynamic Expression Profiles Under Cold Stress in *Madhuca longifolia*

**DOI:** 10.3390/ijms26104713

**Published:** 2025-05-14

**Authors:** Yule Chen, Jiayu Qin, Ziyao Wang, Haoyou Lin, Shuiyun Ye, Jichen Wei, Shuyu Wang, Lu Zhang

**Affiliations:** College of Forestry and Landscape Architecture, South China Agricultural University, Guangzhou 510642, China; llyychen@stu.scau.edu.cn (Y.C.); qjy@stu.scau.edu.cn (J.Q.); helenwang@stu.scau.edu.cn (Z.W.); llucky@stu.scau.edu.cn (H.L.); yeshuiyun@stu.scau.edu.cn (S.Y.); jichen@stu.scau.edu.cn (J.W.); wshuyu@stu.scau.edu.cn (S.W.)

**Keywords:** *Madhuca longifolia*, NAC TFs, low-temperature stress response, multi-omics

## Abstract

*Madhuca longifolia* (*M. longifolia*), a tropical tree valued for its medicinal, nutritional, and industrial applications, exhibits severe sensitivity to low-temperature stress in subtropical regions, particularly during seedling establishment. To address this challenge, this study systematically identified 109 NAC genes in *M. longifolia* and characterized their functional roles in cold adaptation via multi-omics analyses. All NAC proteins were hydrophilic. Key members (e.g., *MlNAC026*, *MlNAC077*, *MlNAC076*) were localized in the nucleus. Phylogenetic analysis grouped them with *ANAC072* (RD26), a homolog involved in leaf senescence and ABA-regulated cold stress responses. The NAC family expanded primarily through segmental duplication. And low Ka/Ks ratios (<1) indicated purifying selection. Promoter analysis highlighted the prevalence of dehydration-responsive DRE and LTR cis-acting elements. Transcriptomic profiling under cold stress identified five continuous differentially expressed genes (*MlNAC026*, *MlNAC040*, *MlNAC059*, *MlNAC077*, and *MlNAC078*) linked to regulatory functions. Homology modeling predicted 3D structures of cold-responsive NAC proteins, and STRING network analysis indicated independent regulatory mechanisms due to the absence of prominent interaction nodes. These findings advance our understanding of NAC-mediated cold tolerance and offer genetic targets to enhance *M. longifolia* resilience in subtropical climates.

## 1. Introduction

*Madhuca longifolia (M. longifolia)*, a traditionally significant medicinal plant in India, is valued for its flowers, seeds, and bark, which exhibit antidiabetic, anti-inflammatory, antimicrobial, and antioxidant properties [1]. Studies highlight the potential of its seed oil in food industries and pharmaceutical development, while its flowers show efficacy in combating anemia and metabolic disorders [2]. Based on our previous study revealing the cold damage symptoms of *M. longifolia* under low-temperature stress after its introduction to southern subtropical China, this paper further investigates the functional roles of *MlNAC* genes in response to cold adaptation challenges. Our earlier findings demonstrated that autumn and winter cold spells (air temperature dropping to ~10 °C) during bud emergence from soil severely impair young shoots and leaves, causing irreversible wilting, growth arrest, and even seedling death despite subsequent temperature recovery [3]. These symptoms align with typical cold injury (>0 °C) mechanisms, including membrane rigidification, protein complex destabilization, and water loss-induced cellular dehydration. Breeding cold-resistant varieties is critical to expanding its cultivation under climate change [4]. Genomic studies targeting cold-responsive genes (e.g., NAC transcription factors) could provide molecular tools for breeding [3].

NAC transcription factors are plant-specific regulatory proteins with a conserved N-terminal DNA-binding domain and a variable C-terminal regulatory region, playing vital roles in growth and stress responses. Under cold stress, NAC transcription factors enhance cold tolerance by regulating downstream genes involved in antioxidant enzyme synthesis and osmotic adjustment. For example, the S-acylation cycle of the transcription factor *MtNAC80* has an impact on the cold stress response of alfalfa (*Medicago sativa*) [5]. Meta-analysis indicates that NAC overexpression (e.g., PbeNAC1, *SlNAC*) activates cold-responsive pathways, stabilizing cell membranes and enhancing reactive oxygen species scavenging [6]. These findings underscore the pivotal role of NAC transcription factors in cold adaptation, with their molecular mechanisms and gene-editing applications holding promise for developing stress-resistant crops.

Members of the NAC family have been identified in various plant species, including *Arabidopsis thaliana* [7,8], *Oryza sativa* (rice) [9], *chenopodium quinoa* [10], *Malus domestica* (apple) [11], *Actinidia* spp. (kiwifruit) [12], *Solanum tuberosum* [13], *Solanum lycopersicum* (tomato) [14], *Zea mays* (maize) [15], *Brassica rapa* (Chinese cabbage) [16], and *Manihot esculenta* (cassava) [17]. Although comprehensive identification and analysis of the NAC gene family have been conducted in model plants, exploration in non-model species, particularly tropical woody trees, remains limited. With the completion of the *M. longifolia* genome sequencing, we identified 109 NAC genes and performed detailed analyses of their phylogenetic relationships, genomic structures, conserved motifs, expansion patterns, and expression profiles under low-temperature stress. Multi-dimensional evolutionary analyses were conducted to elucidate functional roles, while the correlation between cis-regulatory element abundance and cold-responsive expression profiles revealed potential regulatory mechanisms in tropical trees. Three-dimensional homology modeling of key cold-regulated proteins and construction of NAC protein interaction networks further explored their roles in cold stress at the proteomic level [18]. Integrating genomic, transcriptomic, and proteomic data enabled a multi-omics approach to uncover the potential functions of these genes [19].

The prolonged growth cycles of woody plants and the challenges in establishing genetic transformation systems have significantly hindered their improvement. However, our exploration of non-model woody plants should not cease. This study identifies key cold-responsive *MlNAC* genes using integrated transcriptomic and genomic data, offering insights for enhancing cold tolerance in *M. longifolia* and related species.

## 2. Results

### 2.1. Identification of NAC Transcription Factors and Analysis of Protein Physicochemical Properties in M. longifolia

Through the BLAST function in TBtools (v2.110) and the prediction by HMMER 3.0, we finally identified 109 *MlNAC* genes from *M. longifolia* genome. These genes were named *MlNAC001*–*MlNAC109* according to their positions on the chromosomes (Supplementary Table S1). The number of amino acids encoded by *MlNAC* genes ranges from 111 to 1121, with an average of approximately 369. The molecular weight spans from 12,421.07 kDa to 123,393.1 kDa. The isoelectric point varies from 4.46 to 10.7. The aliphatic amino acid index ranges from 46.58 to 85.17, and the average is 65.08. The instability index ranges from 24.26 to 62.95. The instability index indicates that 22 proteins are stable (instability index < 40), while the remaining proteins are unstable. All proteins are hydrophilic, as evidenced by their negative average hydrophilicity values. According to the prediction of subcellular localization, we mainly localized the 109 proteins in the nucleus. A small number is present in the cytoplasm, mitochondria, and other locations (Table 1).

### 2.2. Chromosomal Localization of MlNAC Gene Family Members

**Figure 1:**
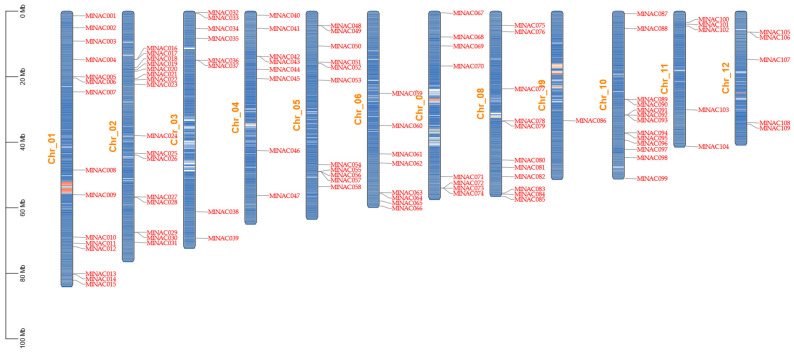
Chromosomal localization of *MlNAC* genes. Chromosome numbers are displayed at the middle point of each chromosome. The scale bars on the left denote genomic length in mega-bases (Mb). Red regions on each chromosome signify high gene density. Blue regions indicate low gene density.

According to the annotation file, we unevenly mapped 109 *MlNAC* genes onto 12 chromosomes (Figure 1). Chromosome 1 contains the largest number of NAC genes, with 16 in total. Chromosome 9 has the fewest *MlNAC* members, with only 1 *MlNAC* gene. It is worth noting that some *MlNAC* genes exist in clusters of two or three, while others exist individually. Some of the genes that exist in clusters are closely related in the phylogenetic tree, and they may jointly perform the same functions (such as *MlNAC42* and *MlNAC43*, *MlNAC73* and *MlNAC74*). In addition, most of the genes on the same chromosome belong to different subfamilies in the phylogenetic tree. It is speculated that the genes on the same chromosome may perform different functions.

### 2.3. Phylogenetic Studies of the NAC Transcription Factor Family in M. longifolia

**Figure 2:**
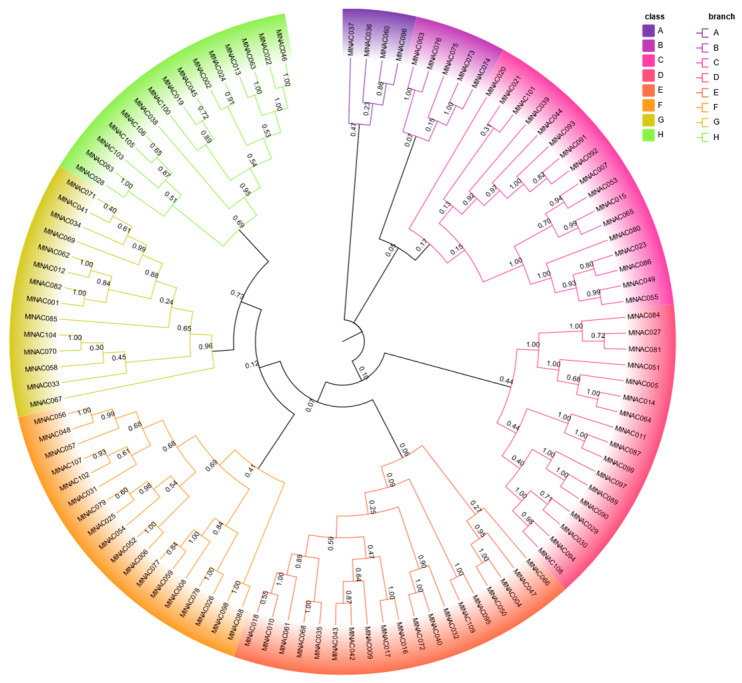
Phylogenetic analysis of *MlNAC* genes. A neighbor-joining (NJ) phylogenetic tree was constructed by MEGA 11.0, using full-length MlNAC protein sequences with 1000 bootstrap replicates. The 109 NAC proteins were classified into eight distinct subgroups (A–H). We highlighted each subgroup by a unique color.

The phylogenetic tree of MlNAC proteins enables us to classify the NAC protein family in *M. longifolia* into eight subfamilies (Figure 2). For the sake of simplicity, these subfamilies are designated as A to H in alphabetical order. Among these subgroups, the largest one is Subfamily E, which contains 19 genes. The smallest subgroup is Subfamily A, which only contains four genes.

**Figure 3:**
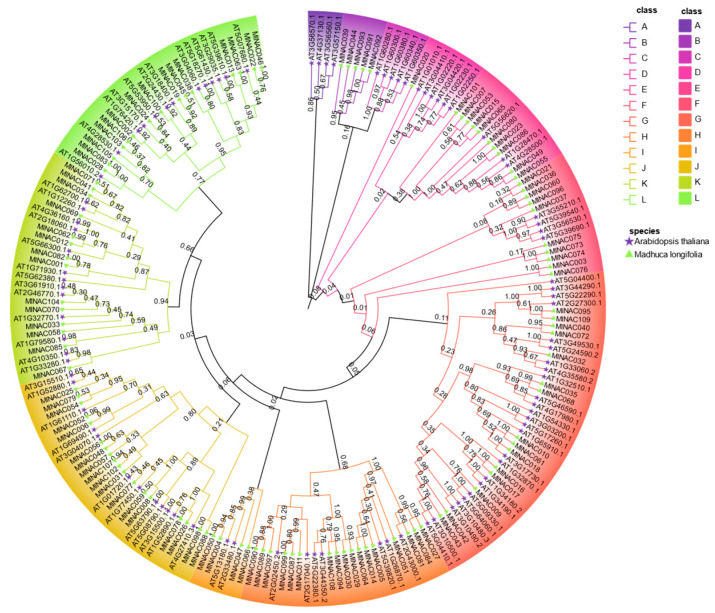
Phylogenetic analysis of *Madhuca longifolia* and *Arabidopsis thalian* NAC genes. An NJ phylogenetic tree was generated by MEGA 11.0, based on full-length NAC gene sequences from both species with 1000 bootstrap replicates. Purple pentagrams preceding *AtNAC* entries denote *A. thaliana* genes. Green triangles preceding *MlNAC* entries represent *M. longifolia* genes. All NAC genes were classified into 12 distinct subfamilies (A–L). Each subgroup was labeled with a unique color.

To further explore the evolutionary interrelationships of *MlNAC* genes, we constructed a phylogenetic tree using 94 NAC proteins from *Arabidopsis thaliana* and the NAC protein sequences of *M. longifolia*. These proteins were jointly classified into 12 groups (Figure 3). Based on the evolutionary relationships, we were able to further infer the functions of the genes in *M. longifolia* that have a close evolutionary relationship with those in *Arabidopsis thalian*.

**Figure 4:**
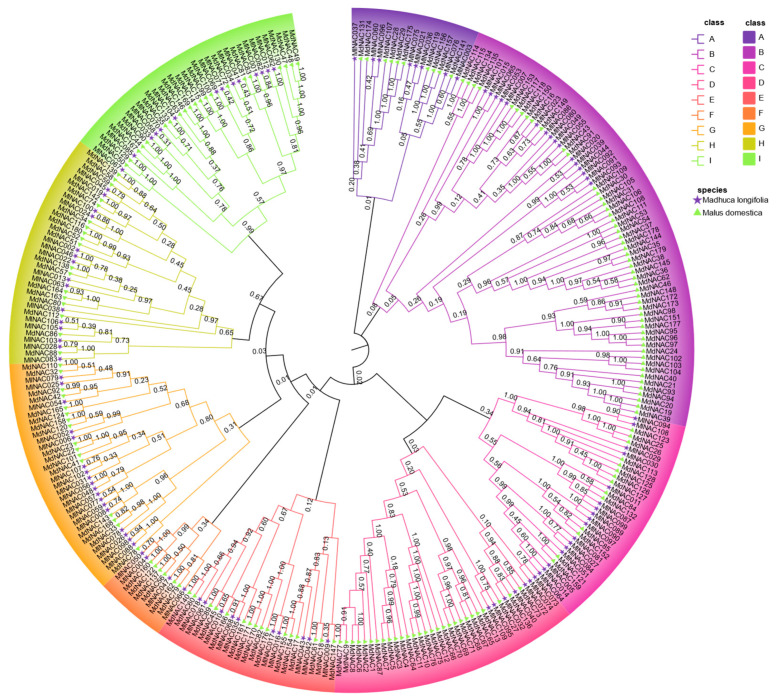
Phylogenetic analysis of *Madhuca longifolia* and *Malus domestica* NAC genes. An NJ phylogenetic tree was constructed by MEGA 11.0, using full-length *MlNAC* and *MdNAC* sequences with 1000 bootstrap replicates. Purple pentagrams preceding *MlNAC* entries denote *M. longifolia* genes. Green markers preceding *MdNAC* entries represent *M. domestica* genes. The 109 NAC genes were classified into nine distinct subfamilies (A–I). Each subgroup was highlighted with a unique color.

Similarly, we also constructed a phylogenetic tree using NAC protein sequences from *Malus domestica* and *M. longifolia*. These proteins were classified into nine distinct groups (Figure 4). Based on the evolutionary relationships, we inferred the potential functions of MlNAC protein closely related to apple NAC genes by referencing the well-characterized roles of apple genes. MdNACs, particularly involved in pigment regulation (e.g., anthocyanin biosynthesis), may provide a useful reference to understand the corresponding MlNAC roles.

**Figure 5:**
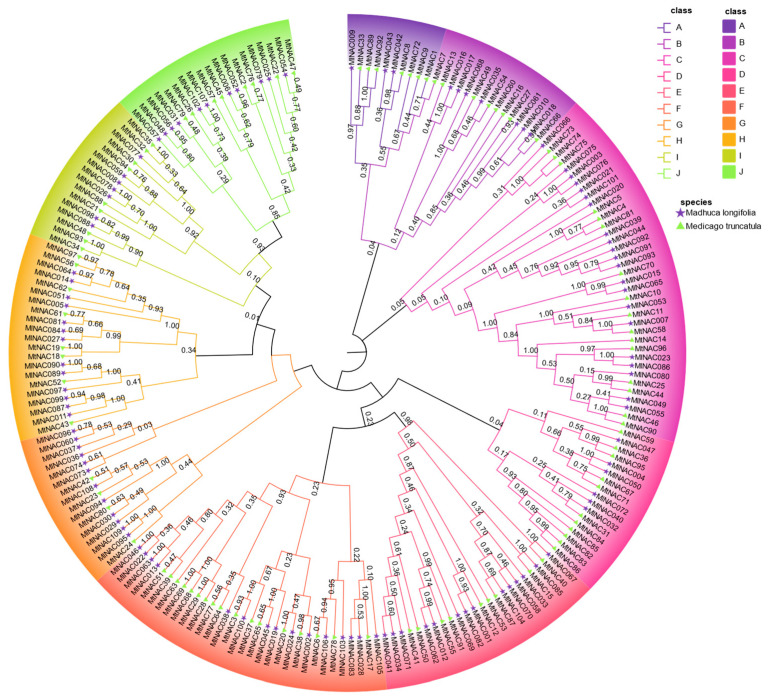
Phylogenetic analysis of *Madhuca longifolia* and *Medicago truncatula* NAC genes. An NJ phylogenetic tree was constructed using NAC gene sequences from both species (1000 bootstrap replications). Purple pentagrams preceding *MtNAC* entries denote *M. truncatula* genes. Green triangles preceding *MlNAC* entries represent *M. longifolia* genes. All NAC genes were classified into 12 distinct subfamilies (A–J), each highlighted with a unique color.

Based on the phylogenetic analysis of NAC protein sequences from *Medicago truncatula* and *M. longifolia*, we categorized these proteins into 11 distinct clades (Figure 5). Although *MtNAC80* and *MlNAC030* were found to share a relatively close evolutionary relationship, the low bootstrap support value (0.63 < 0.7) raises uncertainty regarding the robustness of this clustering. Consequently, whether *MlNAC030* possesses an S-acylation cycle analogous to that of *MtNAC80*—a mechanism critical for regulating cold stress responses in *Medicago truncatula* through nuclear translocation and activation of antioxidant pathways like *MtGSTU1*—remains to be experimentally validated [20,21]. We need to have a further investigation on MlNAC30 (e.g., functional assays and expressional profiling under cold stress).

### 2.4. Analysis of Intraspecific and Interspecific Collinearity of NAC Family Genes in M. longifolia

**Figure 6:**
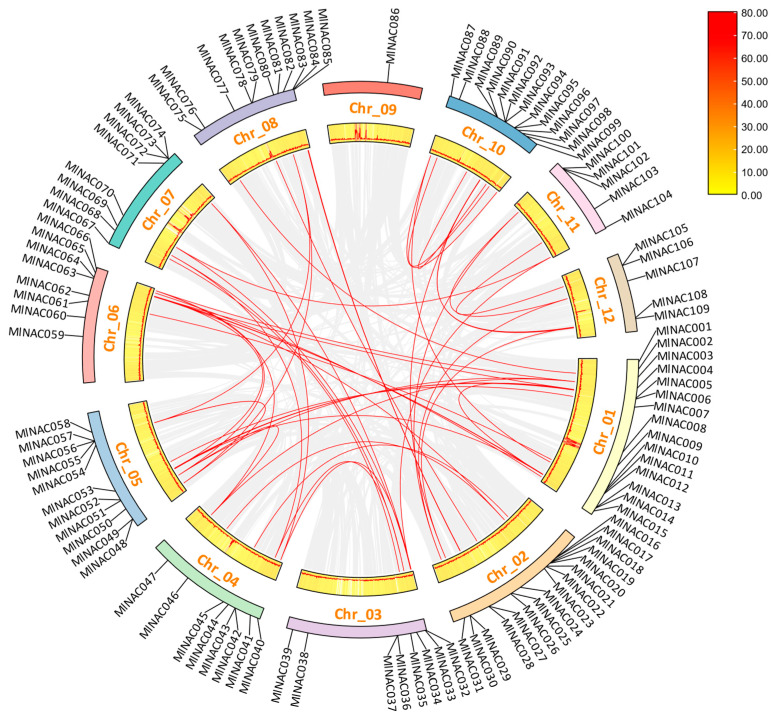
The collinearity analysis of 109 NAC genes is visualized in a circular layout. Gray lines within the inner circle denote intrachromosomal collinear blocks in *M. longifolia*. Red lines highlight replication events associated with *MlNAC* genes. Chromosome names and NAC gene names are, respectively, labeled on the inner and outer sides of each chromosome. Heatmap and lines illustrate gene density distribution. The redder the color, the higher the gene density.

Gene duplication occurs through multiple mechanisms, with segmental duplication, tandem duplication, and whole-genome duplication (WGD). WGD is the primary driver of gene family expansion during evolution [22,23,24,25,26]. These duplication events contribute to the diversification of plant physiological and morphological traits. A comparative analysis of NAC protein sequences in M. longifolia revealed 50 segmental duplication pairs (Figure 6) and six tandem duplication pairs among its 109 *MlNAC* genes. These findings suggest that segmental duplication events played a significant role in the evolutionary expansion of the *MlNAC*s.

The Ka/Ks ratio serves as a pivotal indicator for evaluating evolutionary selection pressures (Table 2). When the Ka/Ks ratio is consistently observed to be less than 1, it strongly suggests that the gene has undergone purifying selection, a process that filters out deleterious mutations to maintain functional stability of the encoded protein.

**Figure 7:**
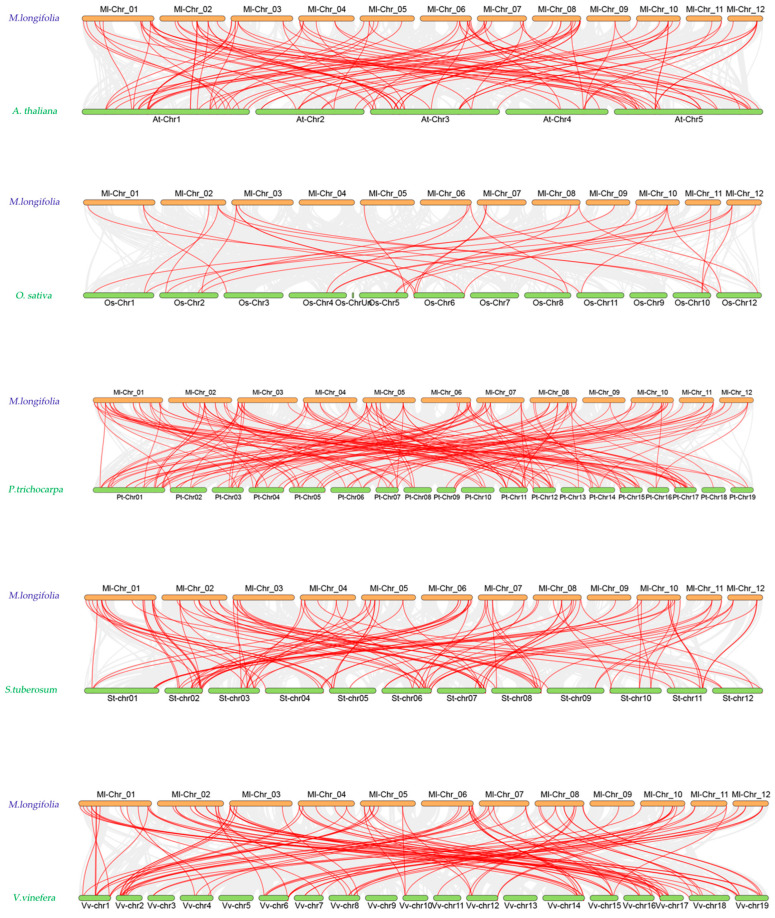
Perform interspecific synteny analysis on *Madhuca longifolia* (*M. longifolia*) with *Populus trichocarpa* (*P. trichocarpa*), *Arabidopsis thaliana* (*A. thaliana*), *Oryza sativa* (*O. sativa*), *Vitis vinifera* (*V. vinifera*), and *Solanum tuberosum* (*S. tuberosum*). The gray lines in the background represent syntenic blocks between *M. longifolia* and the other species. The red lines highlight the syntenic NAC gene pairs.

To further explore the evolutionary relationships of NAC genes across different species, we conducted an interspecific collinearity analysis between *M. longifolia* and *Populus trichocarpa*, *Arabidopsis thaliana*, *Oryza sativa*, *Vitis vinifera* and *Solanum tuberosum* (Figure 7). The NAC family members of *M. longifolia* exhibited the highest number of collinear pairs (162 pairs) with *Populus trichocarpa*, indicating a close evolutionary relationship between these two species. In contrast, *M. longifolia* showed the fewest collinear pairs (33 pairs) with *Oryza sativa*, reflecting their distant evolutionary divergence. Dicotyledonous plants (e.g., Populus *trichocarpa* and *M. longifolia*) share stronger collinearity due to shared whole-genome duplication (WGD) events. Monocotyledons (e.g., Oryza *sativa*) exhibit fewer collinear pairs with dicots. This observation aligns with the broader pattern. Additionally, examining whether a single gene corresponds to multiple homologs could further elucidate functional diversification within the *M. longifolia* NAC family, as gene duplication is a key driver of NAC family expansion and functional innovation.

### 2.5. Motifs and Gene Structures of the MlNAC Transcription Factor Family

**Figure 8:**
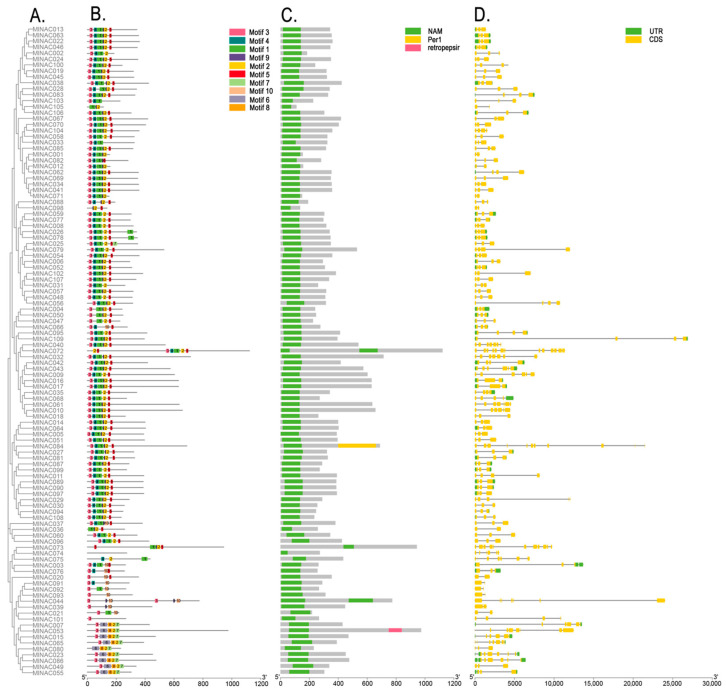
The gene structure of the NAC gene family in *M. longifolia*. (**A**) The neighbor-joining phylogenetic tree of *MlNAC* genes. (**B**) The conserved motifs of *MlNAC* genes. The digit in the box represents the motif number. (**C**) The conserved domain of *MlNAC* genes. (**D**) The exon and intron structure of *MlNAC* genes. The green boxes represent untranslated regions. The yellow boxes indicate the coding sequences. The gray line signify the intron structure of *MlNAC* genes.

NAC proteins possess a conserved NAM domain that is used for DNA binding. This domain is a crucial region for the biological functions of NAC proteins. Therefore, in order to understand the functional differences in NAC proteins and further explore the relationships among the members of the *MlNAC* genes, we analyzed the phylogeny, gene structures, the conserved domain, and conserved motifs of the *MlNAC* transcription factor family (Figure 8). We identified ten conserved motifs among the 109 MlNAC proteins (Supplementary Table S2). The lengths of their amino acids ranged from 8 to 41. Most of the genes contain Motifs 1 to 5, and they are likely to have a certain relationship with the functions of these genes. Although a few genes with close phylogenetic relationships have different motifs, most genes with a relatively close genetic distance possess the same motifs. Notably, certain gene models (e.g., MlNAC101 and *MlNAC74*) lack the conserved NAC domain and display divergent gene structures. These anomalies suggest misannotations that should be corrected using transcriptomic evidence to ensure the genes are correctly identified.

### 2.6. Analysis of Cis-Acting Elements in the NAC Transcription Factor Family of M. longifolia

**Figure 9:**
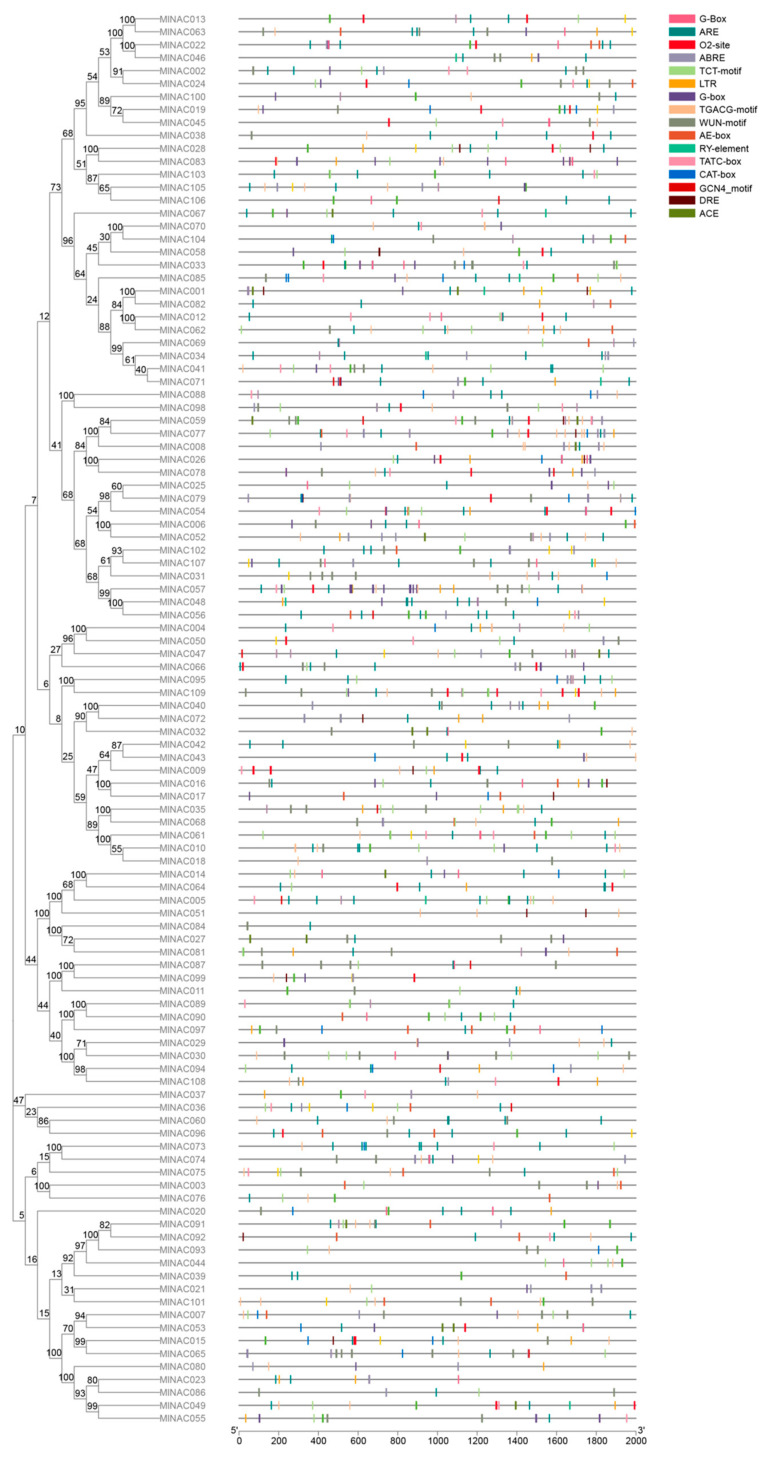
The cis-acting elements of the promoter sequences of NAC genes in *M. longifolia* were predicted. The 17 squares on the right represent the various cis-acting elements of the promoter. And the Neighbor-Joining phylogenetic tree on the left indicates the similarities among NAC genes.

The 2 kb sequence upstream of the *MlNAC* gene was intercepted for cis-acting element analysis. Many cis-acting elements related phytohormone response, light response, stress response and plant development were identified (Supplementary Table S3). Among them, the ones related to the light response were the most abundant. We demonstrated their distribution in the upstream region (Figure 9). We speculate that *MlNAC* transcription factors (TFs) are widely involved in the response of *M. longifolia* to various abiotic and biotic stresses and may possess numerous potential functions in enhancing the stress resistance of *M. longifolia*.

**Figure 10:**
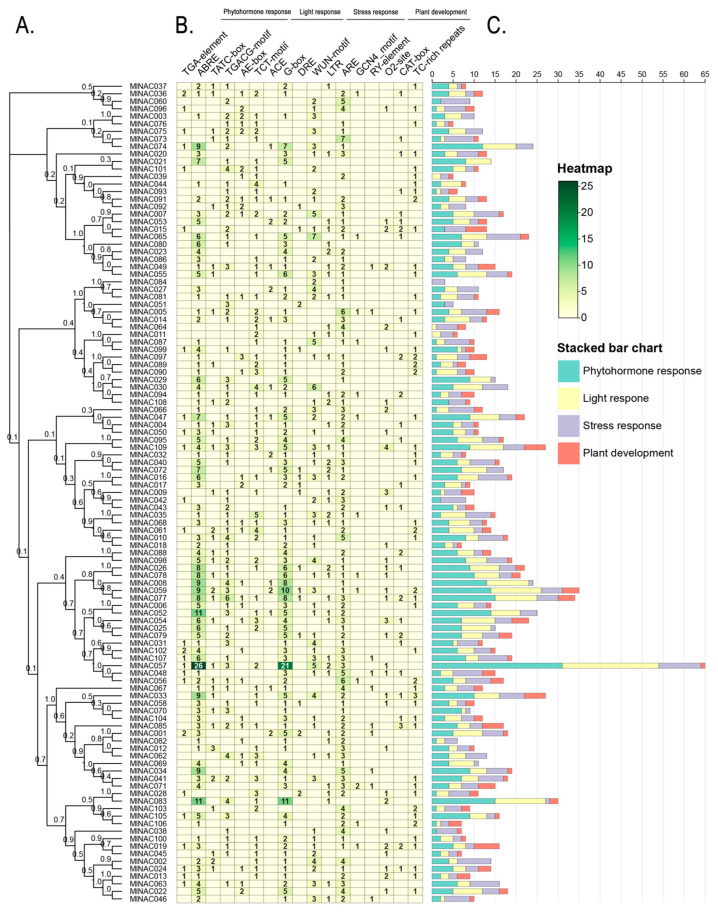
Analysis of the number of cis-acting elements in *M. longifolia*. (**A**) The phylogenic tree of NAC genes in *M. longifolia*. (**B**) Heatmap of the number of cis-acting elements in the corresponding gene. The count of cis-acting elements is in the box. (**C**) The stacked bar chart visualizes the number of four-kind elements. The elements are classified into four groups: Phytohormone, Light response and Plant development.

To further investigate the relationship between cis-acting elements (e.g., LTR and DRE) and gene expression regulation in *M. longifolia*, we conducted statistical analysis of promoter-region elements of each NAC gene (Figure 10). The results revealed that the majority of differentially expressed genes (DEGs) contained these low-temperature-stress-related elements in their promoter regions. It suggests that their potential synergistic roles in transcriptional regulation under cold stress.

### 2.7. Analysis of the Expression Patterns of MlNAC Genes Under Low Temperature

**Figure 11:**
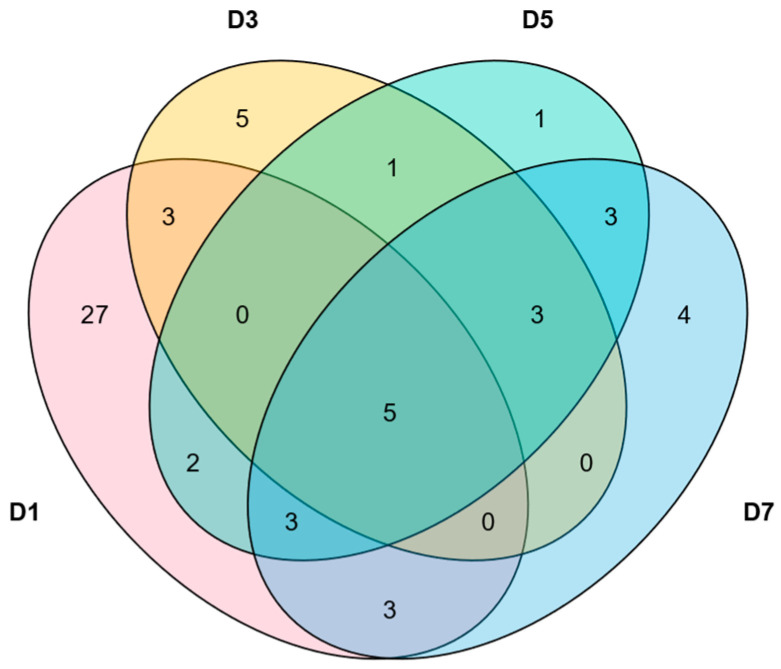
Venn diagram of *MlNAC* genes with differential expression on the 1st, 3rd, 5th, and 7th days (D1, D3, D5 and D7) under low temperature conditions.

Harnessing the acquired transcriptome data, our research cohort meticulously executed a differential expression analysis of the NAC transcription factor family (Supplementary S4–S7). Through simple mathematical statistics, we pinpointed a subset of five genes that manifested consistent differential expression profiles (Figure 11). Under the condition of low-temperature treatment, in comparison with the control group, *MlNAC040*, *MlNAC077*, *MlNAC059*, *MlNAC078*, and *MlNAC026* emerged as the pivotal entities within this differential expression paradigm. Moreover, *MlNAC043*, *MlNAC042* and *MlNAC016* showed differential expression on the 3rd, 5th and 7th days.

We noticed an interesting phenomenon regarding the relationship between *MlNAC077* and *MlNAC078*. These two genes not only possess an exceedingly close evolutionary ancestry but are also spatially located in close vicinity on the chromosome. Such findings strongly insinuate that they may collaborate synergistically to exert a significant impact on the regulatory mechanisms triggered under low-temperature conditions.

**Figure 12:**
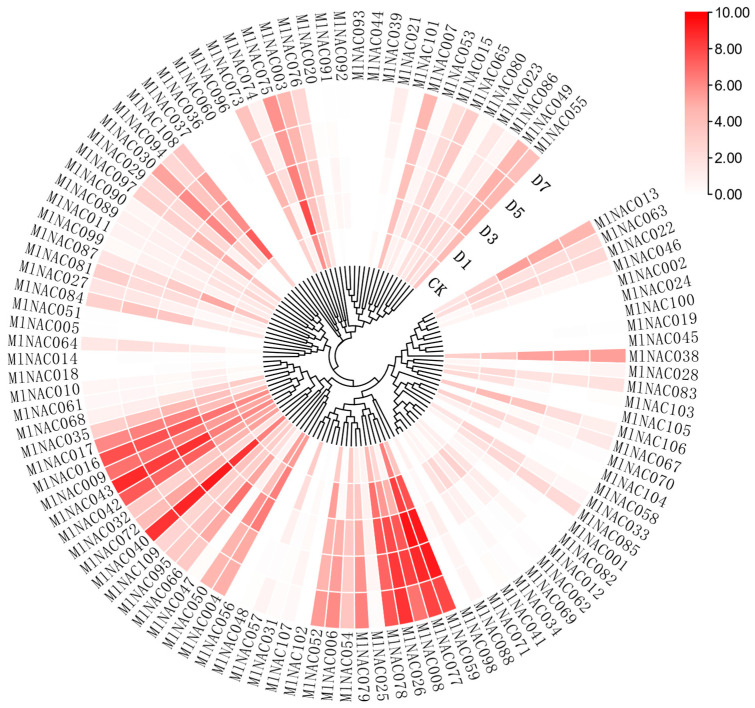
Heatmap of the expression levels of *MlNAC* genes under cold treatment. In the gap in the circle, CK indicates the control group, and D1, D3, D5 and D7 represent exposure to low temperature for 1 day, 3 days, 5 days, and 7 days, respectively. The single figure shows gene relationship and gene expression together by using the phylogenic tree of *MlNAC*s as a core.

To present the expression levels of the transcription factor family in *M. longifolia* under low-temperature conditions more intuitively, we created a heatmap of the expression levels of *M. longifolia* under low-temperature treatment (Figure 12). We found that *MlNAC059*, *MlNAC077*, *MlNAC078*, *MlNAC008* and *MlNAC026* all exhibited significant differential expressions. Moreover, they have a very close evolutionary relationship, and all belong to the F group.

### 2.8. Key Protein Structure Prediction and NAC Protein Interaction Network Prediction

**Figure 13:**
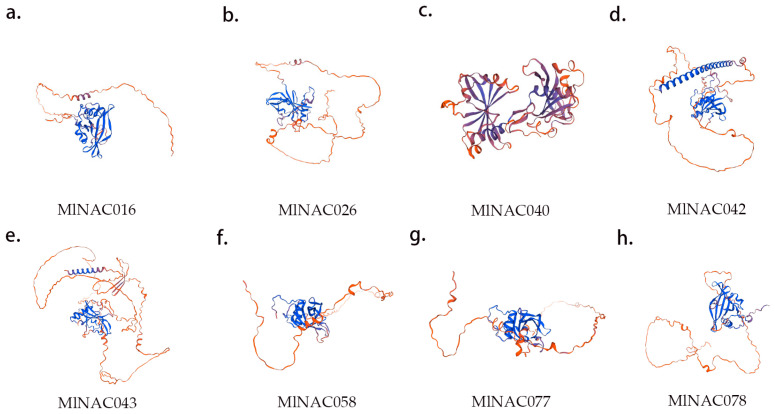
Three-dimensional structure of 8 key MlNAC proteins. (**a**) Three-dimensional structure of the protein in sub-family F, *MlNAC006*. (**b**) Three-dimensional structure of the protein in sub-family E, *MlNAC016*. (**c**) Three-dimensional structure of the protein in sub-family F, *MlNAC26*. (**d**) Three-dimensional structure of the protein in sub-family E, *MlNAC040*. (**e**) Three-dimensional structure of the protein in sub-family E, *MlNAC042*. (**f**) Three-dimensional structure of the protein in sub-family E, *MlNAC043*. (**g**) Three-dimensional structure of the protein in sub-family F, *MlNAC077*. (**h**) Three-dimensional structure of the protein in sub-family F, *MlNAC078*.

The structure of proteins is one of the focuses in the field of bioinformatics. Through structure prediction, we could gain in-depth insights into the functions of proteins, their interactions, and the biological processes. In this study, we utilized the Swiss-model online website to conduct structural prediction on the key MlNAC proteins under cold regulation (Figure 13) (Supplementary Table S8). The research results show that MlNAC proteins within the same subfamily exhibit a high degree of structural similarity, while there are significant structural differences among proteins from different subgroups. Such structural differences are somewhat related to the distinctions in their functions. It is highly likely that the structures of these proteins are closely associated with the functions they perform during the process of cold regulation. In subsequent research, we will focus on the adaptive relationship between protein structure and function and conduct in-depth exploration of the underlying mechanisms.

**Figure 14:**
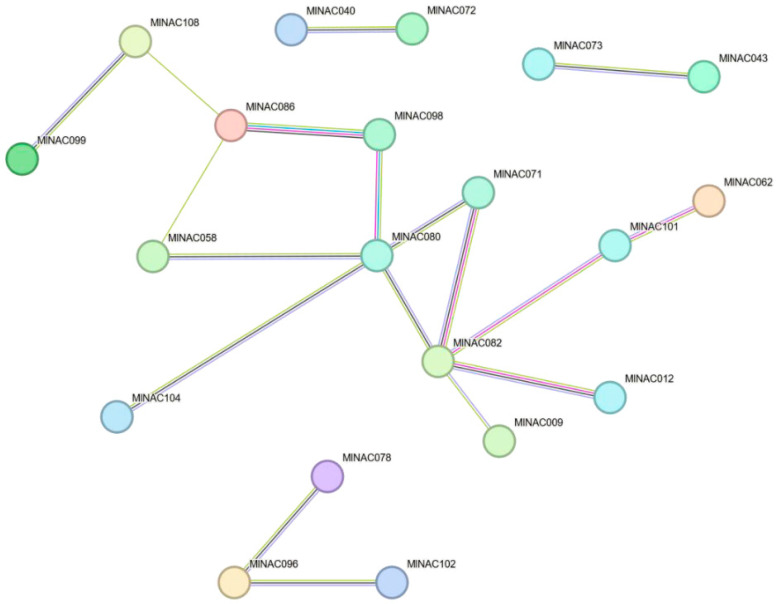
Visualization of the protein–protein interaction of MlNACs using the STRING 12.0 online tool with *Arabidopsis thaliana* as the reference genome. Notably, the proteins (MlNAC058, MlNAC080, MlNAC082, MlNAC086, MlNAC098, and MlNAC101) are present even at high confidence levels. They are more likely to be key nodes.

In the prediction of protein–protein interaction (PPIs) within the *MlNAC* gene family, we implemented a dual-confidence threshold screening strategy (scores 0.4 and 0.7) to enhance prediction reliability (Figure 14). Notably, six core members (*MlNAC058*, *MlNAC080*, *MlNAC082*, *MlNAC086*, *MlNAC098*, and *MlNAC101*) consistently met the stringent 0.7 confidence threshold. It suggests that they may form stable interaction modules (Figure 14). This finding indicates that *MlNAC*s likely mediate signaling transduction and execute diverse biological functions through dynamically organized PPI networks. They may potentially involve the formation of transcriptional regulatory complexes and coordinate activation of downstream target genes. The application of this rigorous confidence threshold (0.7) effectively minimized false positives while retaining high-confidence interactions. It provides reliable candidates for subsequent functional validation studies.

## 3. Discussion

### 3.1. Evolution and Duplication

The NAC transcription factor family represents one of the largest plant-specific regulatory gene families. NAC plays pivotal roles in both biotic and abiotic stress responses [27,28]. In this study, we performed the first genome-wide identification of 109 NAC genes in M. longifolia, exceeding the numbers reported in pepper (61) and hemp (69) [29,30], but smaller than those in Arabidopsis (117) [7,8], rice (151) [9], soybean (151) [31], and maize (148) [15]. Phylogenetic analysis revealed that MlNAC genes exhibited evolutionary conservation. Low Ka/Ks ratios (<1) indicated strong purifying selection [32]. Notably, segmental duplication (50 pairs) dominated over tandem duplication (6 pairs). It suggests that segmental expansion is the primary driver of NAC family diversification in M. longifolia.

### 3.2. Functional Prediction

Given that this study focuses more on the functional insights provided by the phylogenetic tree, it did not perform rigorous grouping of NAC sequences between the target species and other species. This has somewhat affected the robustness of the phylogenetic tree and needs to be further addressed in future research.

Conserved domain analysis demonstrated that NAC subfamilies shared similar gene structures and motif compositions, with motif variations primarily distinguishing different subclades. However, we still need to correct gene structures with obvious errors based on the transcriptome (e.g., MlNAC074 and *MlNAC101*). Strikingly, according to the phylogenic tree, which was constructed by *Madchua longifolia* and *Arabidopsis thaliana*, *MlNAC026* and *MlNAC076* clustered with *Arabidopsis ANAC072* (AT4G27410) and *ANAC019* (AT1G52890) in a highly supported subclade. Given that *ANAC072* regulates ABA/drought/salt/cold responses via antioxidant enzyme activation [33,34,35], and *ANAC019* mediates JA signaling and reproductive development [36,37,38], the conserved clustering and cold-induced expression patterns of *MlNAC026/076* suggest possible potential functions in low-temperature adaptation. Notably, *MlNAC77* showed phylogenetic proximity to *ANAC002* (AT1G01720), which mitigates Cu^2^⁺ toxicity via ROS scavenging in mitochondria/vacuoles [8]. Although lacking high sequence homology, functional convergence warrants investigation.

### 3.3. Compartive Insights

This study investigated the potential functions of MlNAC proteins through systematic comparisons with NAC proteins from apple (Malus domestica) and Medicago truncatula. In apples, MdNAC42 interacts with the key anthocyanin regulator MdMYB10 to significantly promote anthocyanin accumulation in red-fleshed apples. The high homology between MlNAC054 and MdNAC042 suggests a similar regulatory role in anthocyanin biosynthesis in M. longifolia [39]. Notably, MlNAC054 exhibits stable expression under both cold-stressed and non-stressed conditions, indicating its potential independence from low-temperature responses. Another apple NAC transcription factor, MdNAC52, directly binds to the promoters of MdMYB9 and MdMYB11 to activate anthocyanin and proanthocyanidin biosynthesis [40]. Homology-based speculation suggests that MlNAC002, which shares high homology with MdNAC052, may have analogous functions. The consistently low expression of MlNAC002 under cold stress further supports its irrelevance to cold adaptation. Phylogenetic analysis of NAC proteins from Medicago truncatula and M. longifolia classified them into 11 distinct clades (Figure 5). Although MtNAC80 and MlNAC030 showed relatively close evolutionary relationships, the low bootstrap support value (0.63 < 0.7) reduces confidence in this clustering. Thus, whether MlNAC030 shares a mechanism with MtNAC80—such as mediating antioxidant pathways (e.g., activating MtGSTU1) via S-acylation cycling to regulate cold stress responses—requires experimental validation [5]. Transcriptomic data revealed that MlNAC30 displayed a blue color in the control (CK), while its heatmap color shifted significantly in cold-treated groups (D1–D7). Although not reaching strict differential expression thresholds, the distinct heatmap pattern implies potential functional specificity. Future studies should integrate functional assays and expression profiling under cold stress to elucidate MlNAC30’s regulatory mechanisms and potential similarities to MtNAC80.

### 3.4. Cis-Acting Elements and PPI Analysis

Promoter cis-acting element analysis revealed that cold-induced *MlNAC*s harbor cis-regulatory elements associated with cold stress. Intriguingly, *MlNAC077* uniquely possesses a DRE (drought-responsive element) motif absent in *MlNAC076*. It suggests distinct regulatory mechanisms—either local regulation of adjacent genes or long-distance transcriptional control. Classification of cis-elements into four categories (stress, phytohormone, light, and plant development) confirmed *MlNAC*s’ dual roles in growth-regulation and stress adaptation.

From the perspective of protein analysis, we conducted two investigations: protein structure prediction and construction of protein–protein interaction (PPI) networks using STRING. Theoretically, the three-dimensional structure of transcription factors (TFs) determines their function in binding specific cis-acting elements to regulate target gene expression [41,42,43]. This functional execution often requires TFs to form dimers with interacting proteins, which explains our focus on predicting key protein structures. These predictions not only reveal structural features but also lay the foundation for identifying specific binding sites in future studies. PPI network analysis via homology mapping is limited by its reliance on conserved sequence homology for interaction inference, with results inherently speculative. Using two confidence thresholds (0.7 and 0.3), we observed no cold stress-related interactions under stringent thresholds. By relaxing the threshold to 0.3, we identified two intriguing protein pairs: *MlNAC040*-*MlNAC072* and *MlNAC043*-*MlNAC073*. Although these interactions had low confidence scores, transcriptional heatmap visualization revealed sustained upregulation of *MlNAC040* and *MlNAC072* under cold stress, suggesting their collaborative roles in cold adaptation. In contrast, *MlNAC043* and *MlNAC073* showed minimal expression changes, leaving their functional significance in cold response uncertain.

### 3.5. Future Directions

For functional validation, we propose two feasible strategies: (1) establishing *M. longifolia* transformation systems to overexpress *ANAC072*/*ANAC019* homologs (e.g., MlNAC026/076) for phenotypic analysis under drought/cold stress, (2) heterologous expression of *MlNAC026/076/077* in *Arabidopsis* to dissect their cross-species functionality, or (3) conducting detailed predictions of nucleotide-binding sites for cold stress-responsive NAC transcription factors and validate potential dimer-forming protein partners via yeast two-hybrid assays. Given the above predicted functions among these genes, combinatorial overexpression (single/multi-gene vectors) could elucidate their individual function and synergistic functions. This research theoretically proposes gene functions in *M. longifolia*, and we are currently conducting experimental validation.

## 4. Materials and Methods

### 4.1. Plant Material

The fallen mature seeds of *M. longifolia* were collected in Tianhe District (23°11′7.3″ N, 113°21′50″ E), Guangdong Province, China, specifically at the South China National Botanical Garden.

To investigate cold stress responses in *M. longifolia*, we conducted controlled experiments under simulated cold conditions. Naturally shed seeds from maternal plants previously used for DNA isolation were collected for germination. Seedlings were initially cultivated on moist filter paper until embryonic root emergence, then transferred to peat-based substrate under standardized growth conditions matching those described in Section 2.1. Uniform 9-month-old specimens were selected for climate chamber exposure (5 °C, 65% RH, 12 h photoperiod, 17,600 lux). Leaf samples were collected at five timepoints: baseline (CK), and after 1 (D1), 3 (D3), 5 (D5) and 7 (D7) days of cold exposure, with triplicate biological replicates per timepoint. CK serves as a control, providing baseline gene expression without cold stress. D1 (1 day) captures early stress responses as plants activate rapid defenses. D3 (3 days) and D5 (5 days) focus on mid-term regulatory changes during physiological and metabolic adjustments. D7 (7 days) targets long-term adaptation, examining gene expression under prolonged cold stress. Together, these timepoints enable systematic analysis of *M. longifolia* gene expression during cold stress.

RNA extraction employed TRIzol reagent (Invitrogen), followed by quality verification through Agilent 2100 Bioanalyzer analysis and RNase-free agarose gel electrophoresis. Poly(A) + mRNA enrichment used oligo(dT) beads, with subsequent fragmentation and cDNA synthesis performed via NEB Next Ultra RNA Library Prep Kit (NEB #7530). Sequencing libraries were processed on Illumina NovaSeq6000 platforms at Gene Denovo Biotechnology (Guangzhou, China). Paired-end clean reads were used for mapping to the reference genome. This experimental design allows systematic analysis of transcriptional changes during cold acclimation while maintaining consistent genetic background through maternal seed sourcing. The dual RNA quality assessment approach ensures data reliability for downstream expression analyses.

### 4.2. Genome-Wide Identification of the NAC Transcription Factor Family and Prediction of Physicochemical Properties

The genome of *M. longifolia* was assembled by us in a previous study [3]. The genome data of *Arabidopsis thaliana* and AtNAC protein sequences were downloaded from the TAIR database (https://www.arabidopsis.org/, accessed on 20 January 2025) [44]. The Hidden Markov Model (HMM) file for the NAM domain (PF02365) was retrieved from the InterPro website (https://www.ebi.ac.uk/interpro/ accessed on 17 November 2024) [45]. Using the NAM domain, we searched for MlNAC protein sequences with the HMMER 3.0 software with an E-value threshold of 1 × 10^−3^. under an E-value cutoff of 1 × 10^−5^, we used the BLASTP function in TBtools to screen candidate members of the gene family from *M. longifolia* [46]. Considering both the results of the HMMER model and the alignment, we identified 109 NAC proteins. Finally, all candidate *MlNAC* genes were validated using the Conserved Domain Search tool (https://www.ncbi.nlm.nih.gov/Structure/cdd/wrpsb.cgi, accessed on 21 January 2025). We predicted physicochemical properties such as molecular weight (MW) and isoelectric point (pI) using an online website (https://web.expasy.org/compute_pi/, accessed on 21 January 2025) [47]. The subcellular localization of *MlNAC* proteins was predicted using the online website (https://wolfpsort.hgc.jp/, accessed on 21 January 2025) [48].

### 4.3. Phylogenetic Analysis of NAC Proteins in M. longifolia

MtNAC protein sequences were downloaded from a website (https://link.springer.com/article/10.1007/s12298-017-0421-3, accessed on 24 February 2025) [20]. In the MEGA 11 (v11.0.13) [49], we used the Muscle tool with default parameter settings to perform multiple protein sequence alignments on 109 MlNAC of *M. longifolia*. The results of these alignments were then used to construct a phylogenetic tree via the neighbor-joining method, with the p-distance model, a bootstrap value of 1000, and other default parameters, based on which all members of the *M. longifolia* NAC protein family were classified. Similarly, three phylogenetic trees were generated with protein sequences (MlNAC and AtNAC, MlNAC and MtNAC, MlNAC and MdNAC) All phylogenetic trees were beautified using the ChiPlot website (https://chiplot.online/tvbot.html, accessed on 22 January and 24 February 2025) [50], where different subfamilies were distinguished by different colors.

### 4.4. Chromosomal Distribution

We used the annotation file from the *M. longifolia* genome in the TBtools software (v2.110) to obtain the chromosomal localization information of *MlNAC* genes. Then, we employed TBtools to analyze the distribution of *MlNAC* genes on chromosomes and the gene density of each chromosome and to create a corresponding map. In the “Gene Density Profile” function, the “Bin Size” parameter was set to 100 kb, and the remaining parameters were set to their default values.

### 4.5. Collinearity Analysis

To investigate the expansion patterns of the NAC gene family in *M. longifolia*, we performed a self-alignment analysis of the genome using the MCScanX tool in TBtools with an E-value threshold of 1 × 10^−10^. The collinearity results were integrated with gene density profiles and visualized using the Advanced Circos module. For further exploration of NAC family expansion mechanisms, we quantified the numbers of segmentally and tandemly duplicated NAC gene pairs based on collinearity analysis. To assess selective pressures acting on duplicated NAC genes, we calculated the Ka/Ks ratios for these gene pairs using the Simple Ka/Ks Calculator (NG) implemented in TBtools. Gene pairs (with Ka/Ks < 1, =1, or >1) were interpreted as undergoing purifying selection, neutral evolution, or positive selection, respectively. This integrated approach combines collinearity-based duplication detection to elucidate the driving forces behind *MlNAC* family expansion.

To investigate the evolutionary relationships of the NAC gene family in *M. longifolia*, interspecific collinearity analysis was performed using the “MCscanX” tool embedded in TBtools. Genomic data of five species (*Populus trichocarpa*, *Arabidopsis thaliana*, *Oryza sativa*, *Vitis vinifera*, and *Solanum tuberosum*) were retrieved from the Phytozome database (https://phytozome-next.jgi.doe.gov/, accessed on 13 February 2025) [51]. Genome-wide alignment results were processed by “MCscanX” to identify collinear gene pairs between *M. longifolia* and each target species, with an E-value threshold of 1 × 10^−10^. Potential gene duplication events (e.g., tandem or segmental duplications) were examined by filtering collinear regions where a single gene corresponded to multiple homologs. Visualization of syntenic blocks was achieved via the “Dual Systeny Plot” module in TBtools, with customized chromosome order and color schemes to highlight evolutionary patterns. The analysis focused on collinear pair counts and chromosomal distribution patterns to elucidate genomic drivers of evolutionary divergence.

### 4.6. Analysis of NAC Protein Structure and Conserved Motifs

The conserved motifs of NAC proteins were analyzed using the MEME tool (v5.5.7) (http://meme-suite.org/index.html, accessed on 22 January 2025) [52]. The gene structure was analyzed using the TBtools software. Then, the gene structures, conserved motifs and the conserved domain of MlNAC proteins were visualized using TBtools.

### 4.7. Analysis of Cis-Acting Elements of NAC Proteins

Use the “GXF Sequence Extraction” tool in TBtools software to extract the 2 kb upstream sequences of *MlNAC* genes. Subsequently, analyze and compare these sequences with the online PlantCare database (https://bioinformatics.psb.ugent.be/webtools/plantcare/html/, accessed on 24 January 2025) to identify and retrieve the cis-acting elements of the *MlNAC*s [53]. Then, integrate these elements with the phylogenetic tree of *M. longifolia* and visualize them using the “Basic BIOsequence View” tool in TBtools. We used Python (v3.7.6) programming to perform statistical analysis on the number of genetic elements, generated a genetic element matrix, and visualized it using the ChiPlot website (https://chiplot.online/tvbot.html, accessed on 1 May 2025).

### 4.8. Expression Patterns of NAC Proteins Under Low Temperature

Differential expression analysis of RNAs was conducted using DESeq2 between different groups and edgeR between two samples. Genes/transcripts with a false discovery rate (FDR) below 0.05 and an absolute fold change ≥ 2 were identified as differentially expressed. Specifically, DESeq2 was applied for comparisons across distinct experimental groups, while edgeR was utilized for pairwise sample comparisons [54,55]. The analysis employed a negative binomial distribution model to handle count data, with statistical significance determined by the combined thresholds of FDR < 0.05 (to control false positives) and a minimum two-fold change magnitude (to ensure biological relevance). This dual-criterion approach effectively balances statistical rigor and biological significance in identifying differentially expressed genes/transcripts.

First, extract the FPKM expression values of NAC transcription factors in *M. longifolia* under low-temperature stress (control CK, D1, D3, D5, D7 timepoints) from RNA-seq data. Then, organize them into a matrix with genes as rows and samples as columns. Next, import the data into TBtools using the “Heatmap” function, and apply “Log_2_(value + 1)” normalization to the raw FPKM values to eliminate scale differences and enhance the visualization of low-expression genes. At the same time, use the phylogenetic tree of MlNAC proteins to cluster expression profile, which aims to show NAC relationship and expression together. Finally, adjust color gradient and export the map.

### 4.9. Key Protein Structure and NAC Protein Interaction Network Prediction

The 3D structures of MlNAC proteins were constructed by homology modeling using the SWISS-MODEL online tool (https://swissmodel.expasy.org/, accessed on 26 January 2025) [56]. Perform protein–protein interaction prediction for the NAC protein family in *M. longifolia* using the STRING 12.0 tool (https://string-db.org/, accessed on 14 February 2025) with a confidence score threshold of 0.4 and removal of isolated nodes [57].

## 5. Conclusions

This study comprehensively characterized NAC transcription factors in *M. longifolia*. We identified 109 *MlNAC*s distributed across 12 chromosomes. Phylogenetic analysis indicated that *MlNAC*s could be divided into eight distinct sub-families. Expression profiling under low-temperature stress revealed widespread responsiveness of *MlNAC*s to cold stimuli. Notably, five genes—*MlNAC040*, *MlNAC077*, *MlNAC059*, *MlNAC078*, and *MlNAC026*—exhibited sustained differential expression, highlighting their potential functional importance. The 3D structural models of key NAC proteins were predicted. By integrating genomic and transcriptomic analyses, this work establishes a foundation for investigating NAC functions in cold adaptation in *M. longifolia*. These findings hold significant promise for guiding genetic engineering and molecular breeding strategies to enhance cold tolerance in this species.

## Figures and Tables

**Table 1:** Prediction of physicochemical properties and subcellular localization of MlNAC proteins.

Sequence ID	Number of Amino Acid	Molecular Weight	Theoretical pI	Instability Index	Aliphatic Index	Grand Average of Hydropathicity	Subcellular Localization
MlNAC001	154	18,067.56	9.02	30.78	62.01	−0.736	cyto
MlNAC002	184	21,329.25	8.64	38.55	62.55	−0.723	cyto
MlNAC003	263	29,405.18	8.18	43.99	70.08	−0.488	cyto
MlNAC004	240	27,362.78	8.65	43.88	60.88	−0.789	nucl
MlNAC005	389	43,528.92	8.66	59.18	59.46	−0.54	nucl
MlNAC006	293	33,207.45	9.17	32.8	62.25	−0.68	nucl
MlNAC007	429	48,232.66	5.04	50.32	67.67	−0.796	chlo
MlNAC008	317	36,579.04	5.8	55.1	61.32	−0.792	cyto
MlNAC009	602	66,549.72	5.11	41.47	73.22	−0.361	nucl
MlNAC010	657	74,134.14	5.87	51.85	67.84	−0.615	plas
MlNAC011	390	44,480.42	6.19	49.78	72.97	−0.482	nucl
MlNAC012	157	18,572.19	8.66	38.97	62.74	−0.743	mito
MlNAC013	343	38,898.95	5.44	46.91	60.52	−0.678	nucl
MlNAC014	399	44,718.46	6.03	45.34	59.67	−0.603	nucl
MlNAC015	470	52,209.88	4.85	49.96	64.96	−0.683	nucl
MlNAC016	630	70,689.88	4.75	50.07	64.83	−0.647	nucl
MlNAC017	630	70,690.55	4.9	49.07	67.33	−0.588	nucl
MlNAC018	262	30,223.44	5.43	47.5	59.16	−0.858	nucl
MlNAC019	318	35,802.99	6.14	41.55	63.24	−0.615	nucl
MlNAC020	354	38,681.94	4.46	33.94	74.72	−0.421	cyto
MlNAC021	218	24,918.81	4.84	47.88	60.78	−0.753	nucl
MlNAC022	360	40,498.04	8.13	33.3	70.42	−0.523	nucl
MlNAC023	451	50,548.99	6.33	46.38	59.02	−0.908	nucl
MlNAC024	349	38,608.43	7.11	48.73	64.04	−0.494	nucl
MlNAC025	348	38,494.09	7.81	42.29	69.83	−0.63	nucl
MlNAC026	340	38,058.8	8.91	41.23	62.5	−0.586	nucl
MlNAC027	321	36,823.03	6.61	31.9	64.39	−0.856	cysk
MlNAC028	340	37,832.75	6.98	48.35	61.59	−0.518	nucl
MlNAC029	289	33,036.78	5.38	53.9	63.77	−0.684	nucl
MlNAC030	255	29,119.4	6.46	58.44	49.33	−0.908	nucl
MlNAC031	260	29,481.4	8.9	42.33	63.04	−0.669	nucl
MlNAC032	713	79,667.29	5.56	57.45	69.16	−0.651	nucl
MlNAC033	324	36,182.55	5.02	43.57	59.26	−0.8	nucl
MlNAC034	354	41,066.81	6.19	55.27	57.32	−0.891	pero
MlNAC035	341	38,750.11	5.12	48.41	48.53	−0.734	nucl
MlNAC036	258	29,094.2	6.55	45.8	53.64	−0.99	nucl
MlNAC037	380	43,909.44	5.19	56.13	58.71	−1.016	nucl
MlNAC038	423	48,492.12	5.85	45.2	52.03	−0.861	nucl
MlNAC039	447	50,537.86	5.13	48.39	76.51	−0.599	cyto
MlNAC040	539	60,286.63	5.44	40.63	67.46	−0.619	nucl
MlNAC041	357	41,689.57	6.01	53.29	63.05	−0.889	pero
MlNAC042	416	46,467.24	5.57	44.99	75.31	−0.6	chlo
MlNAC043	573	63,561.84	4.56	38.94	72.67	−0.472	chlo
MlNAC044	773	85,627.26	5.91	52.35	73.95	−0.446	nucl
MlNAC045	321	36,140.6	8.32	47.4	67.13	−0.621	nucl_plas
MlNAC046	345	38,756.19	8.66	34.91	73.71	−0.492	nucl
MlNAC047	225	25,293.48	8.96	46.15	63.24	−0.644	cyto_nucl
MlNAC048	310	35,162.77	6.33	41.45	71.71	−0.555	nucl
MlNAC049	337	37,586.41	8.54	41.57	71.1	−0.69	nucl
MlNAC050	246	27,882.26	8.97	43.17	54.63	−0.784	nucl
MlNAC051	395	45,035.59	7.64	45.53	60.99	−0.701	nucl
MlNAC052	307	34,989.1	7.65	34.33	61.01	−0.777	cyto_nucl
MlNAC053	973	108,909.9	5.71	45.2	77.81	−0.561	nucl
MlNAC054	358	39,441.27	7.79	35.24	63.24	−0.649	nucl
MlNAC055	303	33,915.89	6.67	48.77	68.84	−0.76	nucl
MlNAC056	314	35,957.94	6.84	48.63	75.13	−0.624	nucl
MlNAC057	316	35,537.07	6.96	43.92	66.36	−0.552	nucl
MlNAC058	324	36,754.93	5.08	42.15	63.77	−0.741	chlo
MlNAC059	303	35,187.92	7.12	47.26	59.27	−0.753	nucl
MlNAC060	344	39,365.82	5.05	52.97	61.22	−0.802	nucl
MlNAC061	635	72,052.48	5.36	44.37	69.24	−0.613	chlo
MlNAC062	353	40,551.19	5.25	53.57	63.77	−0.775	nucl
MlNAC063	354	40,320.37	5.34	37.02	64.44	−0.626	nucl
MlNAC064	402	45,228.78	6.46	44.77	60.65	−0.607	cyto
MlNAC065	389	43,220.1	5.57	41.79	65.86	−0.74	nucl
MlNAC066	276	31,309.61	9.27	52.02	64.2	−0.704	nucl
MlNAC067	418	47,169.16	7.12	42.9	57.18	−0.877	nucl
MlNAC068	271	31,244.46	8.94	33.21	60.74	−0.65	nucl
MlNAC069	348	40,141.64	5.92	56.3	66.41	−0.791	nucl
MlNAC070	403	45,594.45	6.01	52.55	63.37	−0.831	nucl
MlNAC071	150	17,312.42	8.49	38.25	59.8	−0.808	pero
MlNAC072	1121	123,393.1	4.93	47.12	68.22	−0.622	nucl
MlNAC073	943	104,588.3	4.8	44.69	66.31	−0.674	chlo
MlNAC074	272	30,613.57	5.27	36.7	70.26	−0.526	nucl
MlNAC075	434	47,312.69	5.84	49.08	71.27	−0.54	nucl
MlNAC076	256	28,836.65	8.67	54.68	79.61	−0.308	cyto
MlNAC077	297	33,866.32	6.67	49.76	64.07	−0.669	nucl
MlNAC078	346	38,621.3	8.17	33.99	67.08	−0.575	nucl
MlNAC079	529	58,847.39	9.97	52.83	64.18	−0.792	nucl
MlNAC080	230	26,459.87	9.76	42.92	60.04	−0.957	nucl
MlNAC081	327	36,894.07	8.2	46.76	67.09	−0.654	cyto_nucl
MlNAC082	281	32,135.79	5.55	24.8	63.49	−0.721	cyto
MlNAC083	329	37,165.14	7.93	46.79	59.85	−0.561	nucl
MlNAC084	688	79,660.6	8.62	40.58	85.17	−0.189	plas
MlNAC085	315	36,187.75	6.84	39.11	63.78	−0.714	pero
MlNAC086	475	53,104.02	6.47	47.46	62.25	−0.829	nucl
MlNAC087	288	33,471.35	6.42	39.15	73.82	−0.59	cyto_nucl
MlNAC088	191	21,771.36	4.93	45.15	66.81	−0.57	nucl
MlNAC089	386	43,746.99	6.77	57	65.52	−0.697	nucl
MlNAC090	386	43,742.12	6.81	57	65	−0.688	nucl
MlNAC091	289	33,014.05	5.64	49.48	73.49	−0.762	nucl
MlNAC092	266	29,885.38	5.64	38.25	67.78	−0.759	nucl
MlNAC093	311	35,639.33	5.49	49.19	74.6	−0.662	nucl
MlNAC094	246	27,940.25	7.01	58.59	53.94	−0.78	nucl
MlNAC095	412	45,789.47	6.63	62.95	74.51	−0.592	E.R._plas
MlNAC096	425	48,121.78	6.25	43.38	63.72	−0.82	nucl
MlNAC097	390	44,041.14	6.6	55.27	70.51	−0.613	nucl
MlNAC098	136	15,415.93	5.56	55.69	50.07	−0.828	nucl
MlNAC099	271	31,334.04	6.97	42.29	69.11	−0.616	nucl
MlNAC100	240	27,139.7	9.33	29.86	58.58	−0.668	mito
MlNAC101	266	31,598.32	8.68	45.92	73.61	−0.719	cyto
MlNAC102	383	43,051.05	5.8	44.58	64.73	−0.652	nucl
MlNAC103	226	25,452.48	5.12	44.08	71.15	−0.496	chlo
MlNAC104	358	40,926.24	5.81	48.39	60.75	−0.846	nucl_cyto
MlNAC105	111	12,421.07	10.7	24.26	46.58	−0.636	chlo
MlNAC106	303	34,512.5	6.06	44.6	65.58	−0.684	chlo
MlNAC107	336	38,148.58	5.42	43.98	67.65	−0.739	nucl
MlNAC108	234	26,713.28	8.89	47.81	64.96	−0.63	nucl_plas
MlNAC109	394	44,477.22	7.53	53.14	69.52	−0.608	golg

**Table 2:** Analysis of gene duplication types and Ka/Ks ratios for *MlNAC* duplicate gene pairs.

Gene Name	Gene Name	Ka	Ks	Ka/Ks
MlNAC016	MlNAC017	0.19366	0.54	0.357643
MlNAC056	MlNAC057	0.37887	1.94	0.194812
MlNAC072	MlNAC073	0.26521	0.43	0.613301
MlNAC073	MlNAC074	0.17947	0.22	0.812852
MlNAC092	MlNAC093	0.18383	0.44	0.415911
MlNAC105	MlNAC106	0.36074	0.46	0.779525
MlNAC005	MlNAC014	0.2542	1.85	0.137437
MlNAC004	MlNAC047	0.30535	2.1	0.145468
MlNAC004	MlNAC050	0.10598	0.83	0.128115
MlNAC005	MlNAC051	0.26097	1.13	0.231595
MlNAC006	MlNAC052	0.18814	0.9	0.209433
MlNAC007	MlNAC053	0.14392	0.58	0.247552
MlNAC014	MlNAC051	0.313	1.99	0.15738
MlNAC010	MlNAC061	0.19589	0.89	0.2205
MlNAC013	MlNAC063	0.12992	0.7	0.18503
MlNAC014	MlNAC064	0.1225	0.45	0.27049
MlNAC015	MlNAC065	0.18074	0.63	0.287519
MlNAC004	MlNAC066	0.40389	2.42	0.166574
MlNAC005	MlNAC064	0.25499	1.58	0.161317
MlNAC003	MlNAC076	0.18673	0.44	0.427734
MlNAC011	MlNAC087	0.24609	3.03	0.081343
MlNAC022	MlNAC046	0.11964	0.6	0.199585
MlNAC019	MlNAC045	0.14077	0.76	0.184086
MlNAC022	MlNAC063	0.25031	1.1	0.228258
MlNAC027	MlNAC084	0.14583	0.77	0.189329
MlNAC028	MlNAC083	0.18056	0.64	0.28242
MlNAC026	MlNAC078	0.09175	0.7	0.131836
MlNAC029	MlNAC094	0.32689	2.29	0.142457
MlNAC031	MlNAC102	0.39016	1.71	0.228424
MlNAC031	MlNAC107	0.40584	3.57	0.113673
MlNAC029	MlNAC108	0.34506	1.5	0.230004
MlNAC032	MlNAC040	0.49147	2.36	0.208597
MlNAC034	MlNAC041	0.18919	1.49	0.126941
MlNAC033	MlNAC070	0.19103	1.2	0.158538
MlNAC034	MlNAC069	0.11823	0.87	0.135643
MlNAC035	MlNAC068	0.1636	0.89	0.183472
MlNAC032	MlNAC072	0.6226	1.98	0.314718
MlNAC033	MlNAC104	0.28246	1.95	0.144742
MlNAC047	MlNAC050	0.36597	NaN	NaN
MlNAC047	MlNAC066	0.52199	2.02	0.258471
MlNAC040	MlNAC072	0.34701	0.76	0.45545
MlNAC041	MlNAC069	0.2156	1.8	0.119882
MlNAC048	MlNAC056	0.16141	1.04	0.155259
MlNAC049	MlNAC055	0.12889	0.68	0.189038
MlNAC050	MlNAC066	0.40728	2.88	0.141278
MlNAC051	MlNAC064	0.34092	2.04	0.167518
MlNAC055	MlNAC080	0.26642	1.74	0.152886
MlNAC049	MlNAC080	0.25589	1.97	0.129971
MlNAC070	MlNAC104	0.29509	1.88	0.157362
MlNAC088	MlNAC098	0.13961	1.11	0.125641
MlNAC087	MlNAC099	0.1541	1.49	0.103642
MlNAC089	MlNAC097	0.1766	0.92	0.192336
MlNAC094	MlNAC108	0.17362	1.09	0.159737
MlNAC095	MlNAC109	0.20989	0.67	0.315408
MlNAC103	MlNAC106	0.23434	0.65	0.361058
MlNAC102	MlNAC107	0.1665	0.73	0.226942

## Data Availability

We have already stored the genomic sequence data used in this article in the Genome Warehouse of the National Genomics Data Center at the Beijing Institute of Genomics, Chinese Academy of Sciences/China National Center for Bioinformation. The access number is GWHDTZT00000000. These data are publicly available at https://ngdc.cncb.ac.cn/gwh (accessed on 3 September 2024).

## Supplementary Materials

The following supporting information can be downloaded at: https://www.mdpi.com/article/10.3390/ijms26104713/s1.
